# An attempt at fixing the oversimplification of Nightshades’ (genus *Solanum*) epidermal hair complexity

**DOI:** 10.3389/fpls.2023.1176674

**Published:** 2023-09-28

**Authors:** Sakshi Watts, Rupesh Kariyat

**Affiliations:** ^1^Department of Entomology, University of California, Riverside, Riverside, CA, United States; ^2^Department of Entomology and Plant Pathology, University of Arkansas, Fayetteville, AR, United States

**Keywords:** glandular, stellate, integrated pest management, scanning electron microscopy, herbivory, hindrance

## Introduction

Not just animals and humans, most plants also possess a covering of hairs on their body, also known as *trichomes*, to perform a similar function by protecting plants from harsh environmental conditions. In reality, plant hairs perform much more extensive functions. Plants, especially their leaves, are extensively fed on by many insect herbivores (Southwood, 1961; Lawton, 1983; Janz et al., 2006; Watts et al., 2023). And plant hairs resist the survival and growth of insects on the plants either by hindering their movement or/and feeding. And they are not just of one type, several types of trichomes are present in plants, however, they are primarily classified into glandular or non-glandular, based on the presence or absence of a glandular head. Non-glandular trichomes deter herbivore movement and feeding mainly by acting as spiny structures in the way of their target edible tissue (Kariyat et al., 2017; Kariyat et al., 2019; Kaur and Kariyat, 2020; Kaur et al., 2022). In contrast, glandular trichomes majorly hinder herbivory by producing sticky chemicals in their heads which can trap herbivores, producing herbivore-feeding inhibitors, and volatile chemicals to protect plants through tri-trophic interactions (Weinhold and Baldwin, 2011). Furthermore, there is evidence of the rupture of glandular trichomes by herbivores can be an early alert for plants (Peiffer et al., 2009) leading to the induction of Jasmonic acid regulated defense transcripts. Needless to say, they are diverse, and it is plausible to expect that this diversity has functional consequences.

## Current status of trichome classification

One must wonder ‘*why are classification and nomenclature of trichomes so important?*’ To answer this question, let’s think of the human race without any specific first, middle, and last names, and people are just named as either ‘observers’ (audience) or ‘entertainers’ (performers). If you must call out any of those people, it will get tricky and confusing as the human population will be divided into two subpopulations. Just imagine how much the sense of personal, cultural, familial, and historical connections and importance we would be missing. Just like this imaginary world, trichomes of plants were almost exclusively denoted either as glandular or non-glandular. Previous trichome nomenclature literature from specifically 1940 to 2000 (Luckwill, 1943; Uphof, 1962; Roe, 1971; Payne, 1978; Fahn, 1982; Channarayappa et al., 1992) although provide broad trichome types, lacks the specifications and precise characterization for a specific group of plants (for example, genus *Solanum* (nightshades)). Firstly, Luckwill (1943) provided seven categories of tomato (*Solanum lycopersicum L.*) trichomes that were recognized and named based on their morphological features. Later, Uphof (1962) in their book ‘Plant hairs’ reviewed the advancement of trichome identification and characterization since the very first documentation in the 17^th^ century (Hooke, 2003), but concluded that the trichome classification system so far is inconclusive. Roe (1971) documented a few trichomes of *Solanum* and suggested terminology which accounted for many morphological details of those trichomes. Payne (1978) provided a very wide glossary that can be used to name each trichome type. Fahn (1982) provided various peculiar types of glandular and non-glandular trichomes based on their shapes and perceived functions. Channarayappa et al. (1992) added a few more categories of trichomes in tomato plants that were earlier provided by Luckwill (1943). In addition, numerous other studies focusing on the role of trichomes, because of complications and extra work involved in classification and naming trichomes, continued using the generic names of trichomes. For example, Wagner et al. (2004) discussed new approaches and need for studying trichomes and mentioned that both glandular and non-glandular trichomes have various categories, and these trichomes have different kinds of structures associated with them, but those details were beyond the scope of the study, and only the terms ‘glandular’ and ‘non-glandular’ were used for the most part, similar to some of our past studies (Kariyat et al., 2018; Kariyat et al., 2019). More specifications to hair types in such studies would provide us with a more in-depth functions and role of each trichome type.

## Recent advances in trichome nomenclature

Recently, Watts and Kariyat (2021a) attempted to fill the gap in the trichome characterization and nomenclature literature using *Solanum* because of lab interest in studying plant defenses against insect pests, and the diversity of plants associated with *Solanum* (Knapp et al., 2004). Our research on this quest started by successfully growing a mixture of wild and domesticated *Solanum* plants. Hundreds of pictures of both the abaxial (lower side) and adaxial (upper side) leaf surfaces of all species at a similar age were captured using a Desktop Scanning Electron Microscope (DSEM) using a methodology developed by Watts et al. (2022) to eliminate expensive, and time-consuming multiple steps followed in conventional electron microscopy. For morphology, the magnification of leaf samples varied to obtain higher quality trichome images. A constant magnification of a few samples from each species was used to obtain the density of each trichome type. DSEM had an analytical feature using which we were also able to measure the dimensions of the trichomes by tracing them. We then followed with previous literature (Luckwill, 1943; Uphof, 1962; Roe, 1971; Payne, 1978) for sorting out all the useful terms towards a meaningful nomenclature of trichomes. The entire collection of trichome pictures helped us visualize the overall diversity of trichomes which was not limited to just the species, but between the adaxial and abaxial leaf surfaces, and within each leaf surface as well. To categorize each type, naming each type clearly and distinctly was crucial.

Through this detailed examination, three broad three categories were identified: stellate (star-shaped) non-glandular trichome (without glands), simple (without branches) non-glandular trichome (without glands), and glandular trichome (without branches but with gland/s on the tip of hair). Further, each of these broad categories was finely divided and relevantly named into different types by looking at subtle differences between trichomes. For example, stellate non-glandular trichome (SNT) is a generic name for star-shaped trichomes but with the deeper investigation and careful observation, we could divide stellate non-glandular trichome into SNT with short central ray (shorter central branch compared to the rest of the branches), SNT with long central ray (longer central branch compared to the rest of the branches), bifurcated SNT (two branches originating from a common base), multitangulate (having branches at multiple angles), multiradiate (having multiple branches) SNT, porrect stellate (branches resemble the porrect rays of cacti with multiple horizontal rays and a central ray) and porrect-geminate SNT (similar to porrect-stellate but consists of two whorls of branches one over the other).

**Figure 1:**
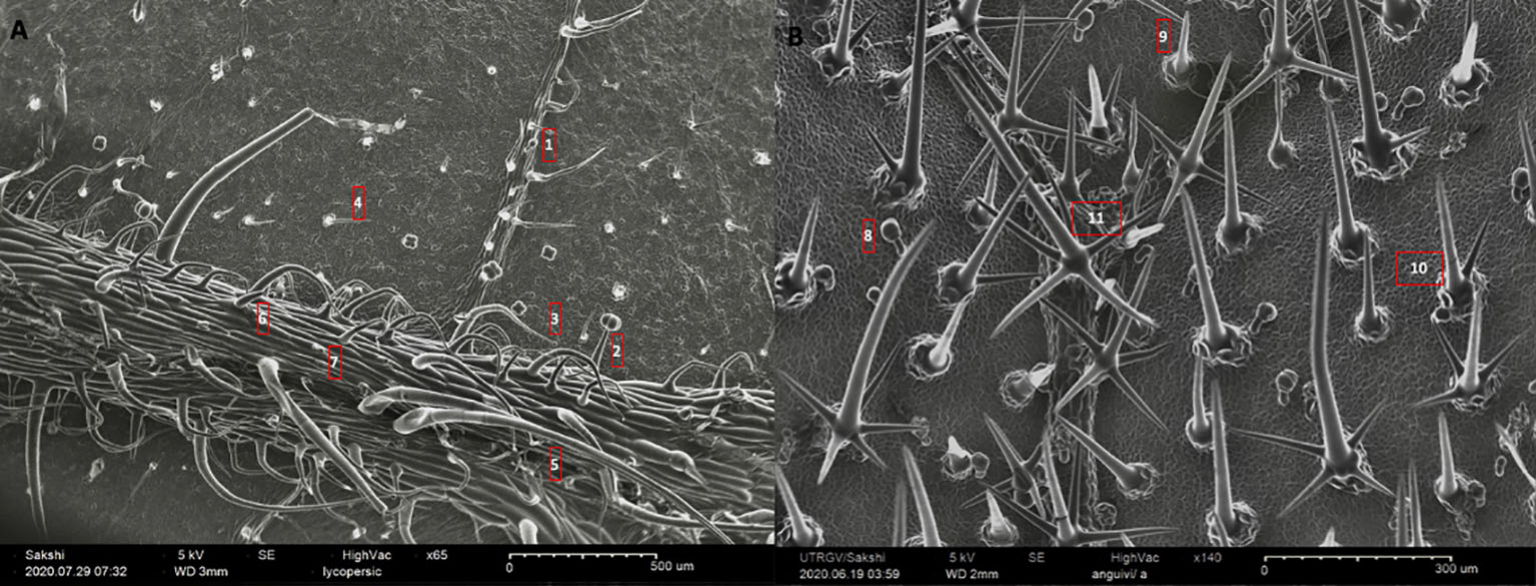
A representative sampling of the morphometric variation for trichomes in nightshades. **(A)** abaxial leaf surface of *Solanum lycopersicum* (65X), and **(B)** adaxial leaf surface of *S. anguivi* (140X). The numbers on the images correspond to the trichome types and their attributes laid out in the **Table 1**.

**Table 1:** The table contains species name, serial number of trichome types in reference to **Figure 1**, and major shape and line art of morphology for each trichome (Adapted from Watts and Kariyat (2021a)).

Species	S. No.	Trichome types (Major shape)	Line art of morphology
*Solanum lycopersicum*	1	Glandular hair with large quadricellular globular head and single stalk cell	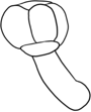
	2	Glandular hair with large quadricellular globular head and single stalk cell	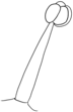
	3	Hooked subulate glandular hair with multicellular jointed stalk and small glandular tip	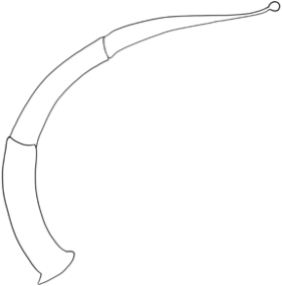
	4	Hooked subulate non-glandular hair	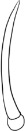
	5	Attenuate non-glandular hair with jointed multicellular stalk	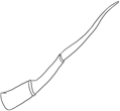
	6	Attenuate basilatus glandular hair with small glandular tip	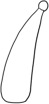
	7	Mamilla non-glandular hair	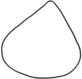
*S. anguivi*	8	Glandular hair with single stalk and neck cell and large quadricellular globular head	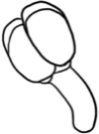
	9	Subulate basilatus non-glandular hair	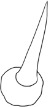
	10	Bifurcated basilatus non-glandular hair with subulate rays (one shorter than the other)	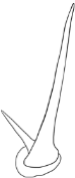
	11	Porrect-stellate multi-radiate non-glandular hair with subulate rays (3-10 in number) with long central ray and pedestal	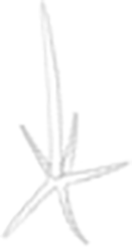

Such thorough categorization was done for two other broad categories as well. Moreover, even if the two trichomes were identical in shape, precise differences in morphology were provided by density and dimension parameters. Additionally, we found some peculiar trichomes types which were very rare or not reported before. With all the data in hand, we came up with two huge tables (one of each leaf surface; Watts and Kariyat, 2021a) containing meaningful names, additional features, line art of morphology, average density, and the average dimension of each trichome type from all species. These tables (Watts and Kariyat, 2021a) are easy to read and easily distinguish between each trichome type in the sequence of “*broad name => finer associated features => its sketch => its density on leaf surface => dimensions”*.

In total, we characterized and named hundreds of trichomes to the finest scale, possibly for the first time. Along with well-defined names and features, our study provides details of the density and dimensions of each trichome type. Overall, it can act as a new catalog of trichomes as it showcases the tremendous variety seen in the *Solanum* genus. A few of the interesting findings in our study are (i) simple non-glandular trichomes have the most diverse types followed by glandular trichomes and compound non-glandular trichomes, and (ii) every species has a unique combination of trichome types and associated density and dimensions on each side of the leaf.

## Discussion

These interesting findings and the overall diversity of trichomes in *Solanum*, leads us to their functional consequences- especially how the variation in density and dimensions of trichomes can be further examined for their correlation with herbivores’ feeding (Watts and Kariyat, 2021b). For example, higher trichome density is generally correlated with reduced herbivory by reducing oviposition (Kumar, 2012) or/and by hindering movement of herbivore (Runyon et al., 2010; Kariyat et al., 2017). However, in a study conducted by Voigt et al. (2007), a positive correlation was found between *Dicyphus errans* (Hemiptera: Miridae) traction force on leaf surface and the trichome density and length. Clearly, there is a need for more in-depth studies which explore the capabilities of plant hair such as defense, considering their physical and chemical properties (especially in case of glandular trichome types).

In the case of glandular trichomes, the entrapping of herbivore by release of sticky substances is an additional advantage other than their hinderance capability for the herbivore. In plants with more than one type of glandular trichome types releasing such sticky substances, the shape and size of those trichomes play an important role as well in measuring precisely, which kind of trichome is more effective in hindering herbivores (Shockley and Backus, 2002). Furthermore, rupturing of the glandular trichomes in some studies have been found to release specific chemicals which helps signal activation of plant defense system (Peiffer et al., 2009). Additionally, the sugars present in the ruptured glandular trichomes have been shown to tag the caterpillars for predation by their natural enemies by modifying their body odor (Weinhold and Baldwin, 2011). Thus, knowing the defense activating structures (trichomes) better can help us know what defense-related chemicals are associated with different glandular trichomes (Gassman and Hare, 2005).

The extensive categorization of trichomes of various species can help us answer some evolutionary questions such as ‘Can the branching pattern of the trichomes explain their functional diversity across the members of each family?, ‘why some species exclusively have glandular or non-glandular trichomes?’, ‘what selection forces decide the combination of different trichome types in a species?’, ‘what mechanisms drive this process, and does a combination of abiotic and biotic stressors play a differential role in deciding trichome traits?’, to mention a few. For example, Beilstein et al. (2006) explored the phylogenetic relationships between 113 species of Brassicaceae family based on the branching patters of the trichomes on each species. Similar studies have been conducted in other species of plants such as Fagaceae (Tschan and Denk, 2012) and Bignoniaceae (Nogueira et al., 2013), etc.

In recent years, many studies have explored the role of non-glandular trichomes in providing physical resistance herbivores (Kariyat et al., 2019; Karabourniotis et al., 2020) and the role of glandular trichomes in providing chemical resistance with direct (Peiffer et al., 2009) and indirect defenses (predators and parasitoids) (Romero et al., 2008), and it has been encouraged to include them in the breeding programs (Snyder and Antonious, 2011; Glas et al., 2012). For example, de Queiroz et al. (2020) screened 18 genotypes of soybean for the antixenosis (non-preference; Kogan and Ortman, 1978) to armyworm moth, *Spodoptera cosmioides* (Lepidoptera: Noctuidae), and found three genotypes in which antixenosis is mediated by trichome density and leaf color. By using Watts and Kariyat (2021a) for similar studies, the specificity for targeting a trichome type can be increased to a greater extent with easier documentation, which ultimately help in the resistance breeding programs. The breeding programs usually lead to the incorporation of the selected genotypes with desirable defense traits, and has the potential to reduce over reliance on chemical insecticides- thereby being an integral part of the integrated pest management programs (Tortorici et al., 2022).

Taken together, now trichomes do not have to be vaguely categorized only as glandular or non-glandular type, unlike the unnatural and misshaped fictional human world of entertainers and observers. Earlier trichomes were originally believed to be evolved for abiotic stresses, but now have much more diversification and functional importance to offer. This study can help and guide phylogeneticists and ecologists to explore the path to find their importance even further. Furthermore, the specific importance that might be associated with each trichome type can be investigated to help entomologists look into their potential for integration in integrated pest management programs, and plant breeders to breed hardy plant varieties.

## Author contributions

SW designed and wrote first draft. RK revised and added tables and figures. SW and RK did further revisions. All authors contributed to the article and approved the submitted version.
